# Sequence Alignment Reveals Possible MAPK Docking Motifs on HIV Proteins

**DOI:** 10.1371/journal.pone.0008942

**Published:** 2010-01-28

**Authors:** Perry Evans, Ahmet Sacan, Lyle Ungar, Aydin Tozeren

**Affiliations:** 1 Genomics and Computational Biology and Department of Computer and Information Science, University of Pennsylvania, Philadelphia, Pennsylvania, United States of America; 2 Center for Integrated Bioinformatics, School of Biomedical Engineering, Science and Health Systems, Drexel University, Philadelphia, Pennsylvania, United States of America; Institute of Infectious Disease and Molecular Medicine, South Africa

## Abstract

Over the course of HIV infection, virus replication is facilitated by the phosphorylation of HIV proteins by human ERK1 and ERK2 mitogen-activated protein kinases (MAPKs). MAPKs are known to phosphorylate their substrates by first binding with them at a docking site. Docking site interactions could be viable drug targets because the sequences guiding them are more specific than phosphorylation consensus sites. In this study we use multiple bioinformatics tools to discover candidate MAPK docking site motifs on HIV proteins known to be phosphorylated by MAPKs, and we discuss the possibility of targeting docking sites with drugs. Using sequence alignments of HIV proteins of different subtypes, we show that MAPK docking patterns previously described for human proteins appear on the HIV matrix, Tat, and Vif proteins in a strain dependent manner, but are absent from HIV Rev and appear on all HIV Nef strains. We revise the regular expressions of previously annotated MAPK docking patterns in order to provide a subtype independent motif that annotates all HIV proteins. One revision is based on a documented human variant of one of the substrate docking motifs, and the other reduces the number of required basic amino acids in the standard docking motifs from two to one. The proposed patterns are shown to be consistent with *in silico* docking between ERK1 and the HIV matrix protein. The motif usage on HIV proteins is sufficiently different from human proteins in amino acid sequence similarity to allow for HIV specific targeting using small-molecule drugs.

## Introduction

ERK1 and ERK2 mitogen-activated protein kinases (MAPKs) have been shown to increase HIV infectivity by phosphorylating a subset of HIV proteins [1]. Prior to HIV replication, the HIV structural protein matrix (MA) must be phosphorylated by ERK2 to allow the HIV preintegration complex to translocate to the nucleus, where viral replication can proceed [2]. Viral replication may also require the phosphorylation of the HIV viral infectivity factor (Vif) by ERK1 or ERK2 [3]. Nef and Tat have been shown to induce the ERK MAPK cascade [4], [5]. ERK1/2 has been shown to phosphorylate HIV Nef, Rev, and Tat in vitro [1], but the roles of these phosphorylation events in HIV infectivity remain unknown [6]. The inhibition of MAPK phosphorylation has been shown to decrease HIV infectivity, indicating that MA and Vif MAPK-directed phosphorylation events would make good drug targets [1], [2].

Most MAPK drugs prevent MAPKs from interacting with ATP by blocking the conserved ATP binding site. The use of ATP binding site in drug development raises concerns about these drugs' lack of specificity [7]. An ERK inhibitor, FR180204, was deemed to selectively inhibit ERK1/2 when it failed to inhibit seven other kinases [8]. However, with nearly 500 kinases left to test [9], specificity is still a concern. New research has suggested more specific means of inhibition may be achieved by preventing MAPK substrate docking [10], [11]. Docking events are guided by docking motifs on substrate proteins that interact with counter-regions on MAPKs [12]. Multiple consensus MAPK substrate docking patterns have been proposed for eukaryotes, although some exceptions to these patterns do exist [13]. Recently, small-molecule inhibitors that target calcineruin phosphatase activity by disrupting docking showed success [14]. With knowledge of docking sites on HIV, small-molecule inhibitors might be developed to disrupt HIV protein phosphorylation by MAPKs, preventing HIV replication. However, care must be taken when designing HIV drugs because of strain diversity.

HIV strains are classified hierarchically, starting with three groups: major (M), outlier (O), and non-major and non-outlier (N). Group M, which is the most common, has been divided into nine subtypes, or clades: A, B, C, D, F, G, H, J and K. Recombinant forms of group M subtypes have been identified. For instance, 01_AE is a combination of subtypes A and E that is circulating in Southeast Asia [15]. Five subtypes and two recombinants are present in at least 2.5% of the world population, making subtype diversity an issue for drug and vaccine design. HIV subtype has been found to influence transmission and disease progression [15]. The presence and absence of short motifs on HIV proteins has been correlated with patient response to certain therapies [16], indicating that differential docking site usage among strains should be considered when designing drugs.

In this study, we examine ERK1/2 docking with HIV proteins from a drug design perspective using multiple alignments of HIV proteins classified according to subtype. We find that only Nef has a consensus MAPK docking site pattern on the most common HIV strains (A1, B, and C). Some of the most frequently observed subtypes of HIV proteins MA, Tat, and Vif are missing the docking pattern most often observed in eukaryotic MAPK substrates, whereas Rev does not show the docking pattern on any strains. To explain MAPK docking with HIV proteins in a strain independent manner, we impose slight revisions on the regular expressions of previously annotated docking motifs. One such revised motif is present in all major subtypes of HIV proteins known to be phophorylated by ERK1/2, and is statistically enriched among the substrates of ERK1/2. The use of *in silico* docking indicates the plausibility of the candidate motifs as HIV protein docking sites for ERK1. Our results provide a first step towards identifying the docking site motifs on HIV proteins and await experimental verification.

## Results

### Consensus MAPK Docking Sites on Human Proteins

MAPK docking site sequences, found in most eukaryotic MAPK substrates, are presented as two distinct patterns, dubbed LIG_MAPK_1 and LIG_MAPK_2, by the Eukaryotic Linear Motif (ELM) Resource [13]. The LIG_MAPK_2 pattern was not considered in this analysis because it was not found to be enriched in human ERK1/2 substrates, and it was not expressed by the HIV proteome (data not shown). The LIG_MAPK_1, or D-site, pattern has two functional regions: a string with two or three basic residues and a chain of alternating hydrophobic residues [12]. One to six residues maintain distance between these regions, helping them interact with distinct regions on MAPKs [17]. The basic component of the motif interacts with MAPKs at a patch of acidic residues, called the common docking (CD) site, while the hydrophobic region of the D-site interacts with a hydrophobic groove close to the CD site [18]. The ELM Resource describes one version of the D-site (*Da*), while the current literature contains another frequently observed MAPK docking motif (*Db*), with a regular expression similar, but not identical, to that of *Da* [12]. Both of the *Da* and *Db* motifs have the same biochemical foundations (Table 1). The regular expressions of docking motifs *Da* and *Db* were kept inclusive to account for MAPK induced phosphorylation in multiple eukaryotic species. Nonetheless, these motifs can serve as starting templates for the discovery of HIV sequences involved in docking to ERK1/2.

**Table 1:** MAPK docking pattern hits on human proteins.

Motif	Pattern	Phos(%)	ERK(%)	p-val
**Da**	[KR]{0,2}[KR].{0,2}[KR].{2,4}[ILVM].[ILVF]	558 (43)	56 (45)	0.299
**Db**	[KR]{2,3}.{1,6}[ILVM].[ILVF]	513 (39)	51 (41)	0.348
**Da/b**	-	620 (48)	69 (56)	0.030
**Dc**	[KR].{2,6}[ILVM].[ILVF]	841 (65)	92 (75)	0.007
**Dd**	[KR].{1,3}[KR]{2}	694 (53)	70 (57)	0.231

Using each of the MAPK docking site patterns, we scanned phosphorylated substrates in dbPTM. We show the number of substrates with pattern matches as well as results for ERK1/2 substrates. We used Fisher's test to calculate a p-value for the enrichment of pattern hits on ERK1/2 substrates compared to all other phosphorylated proteins. The standard docking site patterns, *Da* and *Db*, were not enriched on ERK1/2 substrates, but the union of these patterns was enriched. *Dc*, but not *Dd*, was found to be enriched on ERK1/2 substrates.

To determine the use of docking sites in the human proteome, we scanned proteins with documented phosphorylation sites [19] and ERK1/2 substrates [19] with the *Da* and *Db* docking site patterns, filtering out pattern hits falling in Pfam domains [20] in a manner similar to the one used by the ELM Resource. The results presented in Table 1 indicated that the *Da* U *Db* pattern was enriched on ERK1/2 substrates relative to all phosphorylated substrates (p<0.03). This statistical enrichment provided evidence supporting the validity of the *Da* U *Db* pattern as the MAPK docking site motif among human proteins.

### MAPK Docking Sites on HIV Proteins

**Figure 1:**
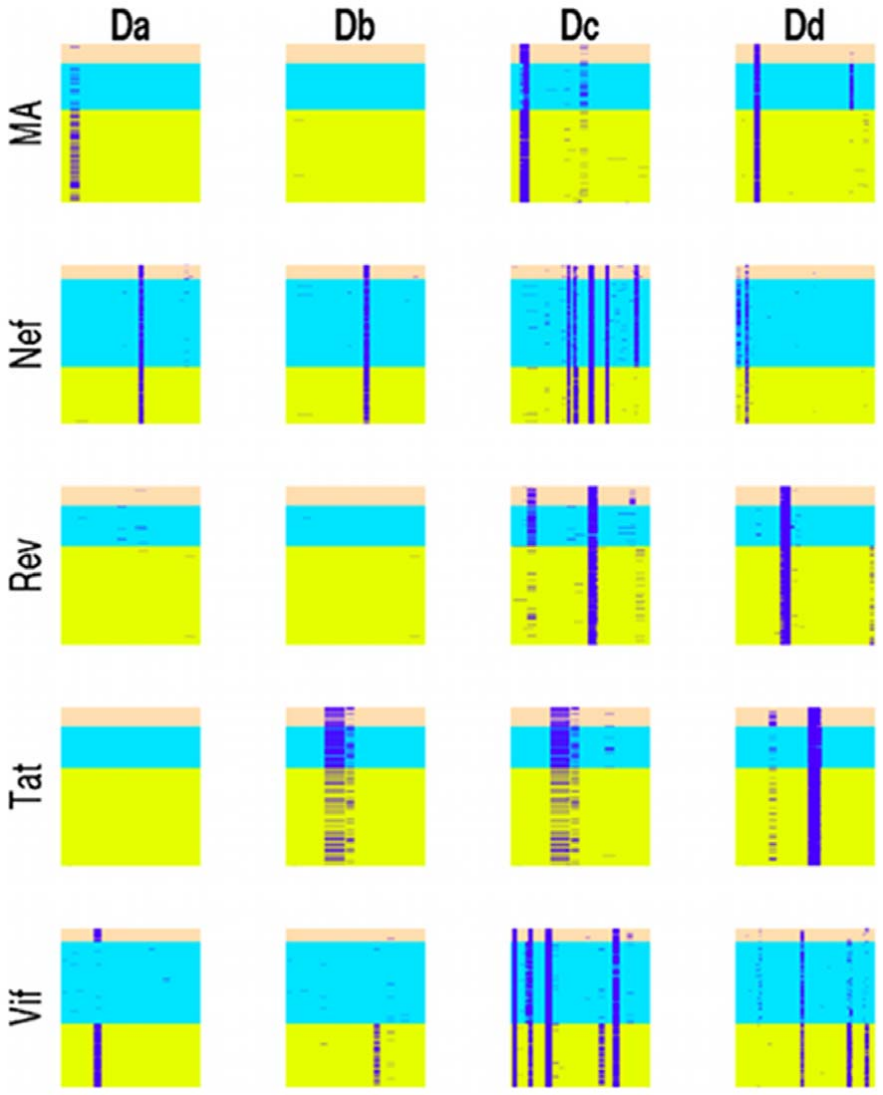
D-site pattern hits on HIV proteins. Hits for the standard MAPK docking sites, *Da* and *Db*, and the proposed MAPK docking sites patterns, *Dc* and *Dd*, are annotated in purple on multiple sequence alignments of HIV proteins MA, Nef, Rev, Tat, and Vif. Subtypes in each alignment are represented by different colors: A1 is pink, B is blue, and C is green. Note that motif annotations occur in roughly the same position within a virus subtype.

Since ERK1/2 substrates were statistically enriched with MAPK docking motifs, we hypothesized that the presence of these docking sites on most sequences of HIV proteins MA, Nef, Rev, Tat, and Vif would explain their reported phosphorylation by ERK1/2. Therefore, we searched for the MAPK docking site patterns on HIV sequences gathered from the Los Alamos National Lab (LANL) HIV Sequence Database (http://www.hiv.lanl.gov/), which contains thousands of sequences spanning multiple subtypes and recombinant forms. In this analysis, we considered HIV strains with at least 50 sequences for all HIV proteins known to interact with ERK1/2, leaving three strains to consider: A1, B, and C. These subtypes are responsible for the majority of the HIV infection around the globe [21]. Figure 1 shows the *Da* and *Db* motif annotations on multiple sequence alignments of HIV proteins, and Table 2 shows the percentages of HIV subtype sequences with docking site matches. The results showed a subtype dependence for the annotations of the *Da* and *Db* patterns along HIV proteins.

**Table 2:** MAPK docking pattern hits on HIV proteins.

A(Da)					B(Db)				
VP	A1	B	C	Total	VP	A1	B	C	Total
**MA**	6	14	50	34	**MA**	1	1	1	1
**Nef**	97	89	98	93	**Nef**	96	89	91	90
**Rev**	1	9	1	3	**Rev**	0	0	1	0
**Tat**	0	0	0	0	**Tat**	67	91	44	59
**Vif**	92	6	97	50	**Vif**	1	5	61	27

C(Dc)					D(Dd)				
VP	A1	B	C	Total	VP	A1	B	C	Total
**MA**	95	96	96	96	**MA**	95	99	97	97
**Nef**	100	100	100	100	**Nef**	60	92	86	87
**Rev**	100	99	96	97	**Rev**	100	100	100	100
**Tat**	69	93	45	61	**Tat**	100	99	100	99
**Vif**	100	100	100	100	**Vif**	92	87	100	92

We searched sequences of HIV proteins using the four MAPK docking site patterns in Table 1. Here we present the percentages of HIV subtype sequences with these docking site patterns. The *Da* and *Db* patterns were found on the majority of Nef sequences, but they were missing from some subtypes of the other HIV proteins. The *Dc* pattern occurred on the majority of MA, Nef, Rev, and Vif subtypes. The *Dd* motif had hits on most sequences of all HIV proteins.

More than 90% of Nef sequences had the *Da* motif regardless of subtype, but this motif was absent on most Tat and Rev sequences. Vif subtypes A1 and C, but not B, expressed the *Da* motif. On the other hand, the *Db* motif was present on Nef, Tat, and subtype C of Vif, but was absent on MA and Rev. The *Da* and *Db* motifs occupied different spatial positions along the HIV proteins. The data shown in Figure 1 suggested Nef was the only HIV protein for which phosphorylation by ERK1/2 could be explained by a standard docking site.

Next we revised the *Da* and *Db* regular expressions in an attempt to find a motif that would be present on all major subtypes of HIV proteins known to interact with human ERK1/2. We looked at sequences of virus proteins without a docking motif coincident with the spatial position of the standard motifs. Specifically, we looked at regions along MA and Vif that aligned with the *Da* motif, as well as regions of Tat and Vif that aligned with the *Db* motif (Figure 1). The absence of the *Da* motif in subtypes A1 and C of MA and subtype B of Vif was caused by a missing basic residue. The absence of *Db* in some subtypes appeared to be due to mutated hydrophobic residues. Taking clues from these perturbations, we designed a new regular expression for a candidate MAPK docking motif along HIV proteins (Table 1), and represented this motif with the symbol *Dc*. The motif *Dc* turned out to be present on HIV proteins Nef, Rev, Tat, Vif, and MA in a relatively strain independent manner (Figure 1).

We also considered whether or not an infrequently observed MAPK docking motif among human proteins could serve as an HIV strain-independent MAPK docking site. The docking site for MAPK with thyroid hormone receptor-beta1 (TRβ1) is KGFFRR. The motif is known to be fully functional, and yet it is missing the hydrophobic portion of the *Da* and *Db* motifs [22]. Furthermore, mutational studies showed that only the first and final two basic residues were required for docking, yielding the pattern KXXXRR, where X represents any amino acid [22]. Scanning this motif along the HIV proteome provided new hits, but did not have sufficient coverage along MA, Rev, Tat, and Vif subtypes. We expanded this pattern to include all basic residues in the first and final two amino acids, and allowed variation in the distance between the basic components, resulting in motif *Dd*, with the regular expression given in Table 1. We used this new pattern to scan multiple alignments of HIV proteins, and found hits on the majority of sequences for all HIV proteins known to interact with MAPK (Figure 1, Table 2).

**Figure 2:**
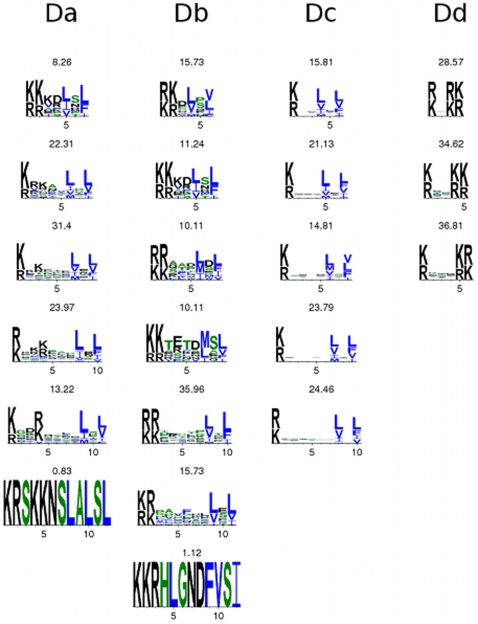
Human sequence logos. For the four MAPK docking sites in the study, we show sequence logos for hits on human proteins. The motifs used here allowed matches with varying lengths. The percentage of motif instances of a certain length is shown above each logo.

**Figure 3:**
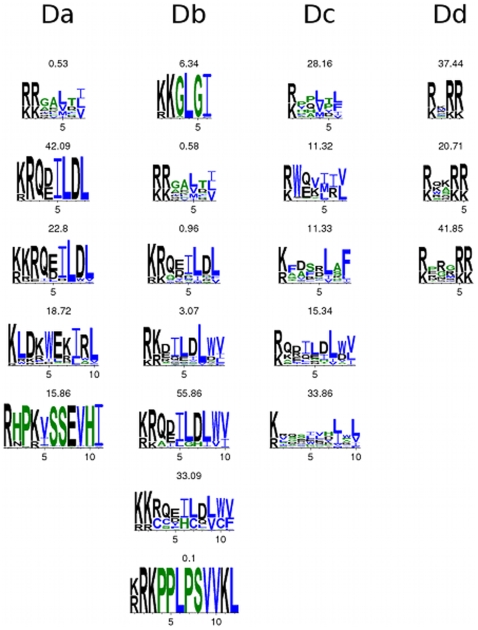
HIV Sequence logos. For each MAPK docking site motif, we gathered all hits on all HIV proteins and constructed sequence logos for hits with the same length. The percentage of motif instances of a certain length is shown above each logo.

The usage of the docking motifs in terms of sequence can be different in human and HIV proteins, and this could best be observed by constructing sequence logos from the motif hits on human (Figure 2) and HIV (Figure 3) proteins known to be phosphorylated by ERK1/2. It was clear from Figures 2 and 3 that the residue usage for motifs *Da*, *Db*, and *Dc* was similar because all motif hits had basic residues followed by hydrophobic residues. This similar biochemical foundation explained why the candidate docking motif *Dc* was coincident with *Da* or *Db* in the HIV proteome. Our computations based on Fisher's exact test showed the *Dc* motif to be statistically enriched on ERK1/2 binding partners (Table 1). The fact that the *Da*, *Db*, *Dc* motif hits on HIV proteins allow less variation in the spacing residues makes it possible to target these regions with small-molecule drugs while preserving host ERK1/2 activity. The *Dd* motif had more or less the same residue usage in human and HIV proteins. This motif is a simple one, and was not statistically enriched among ERK1/2 substrates (Table 1).

### Candidate Docking Motifs on the HIV Protein Matrix Supported by Structures

**Figure 4:**
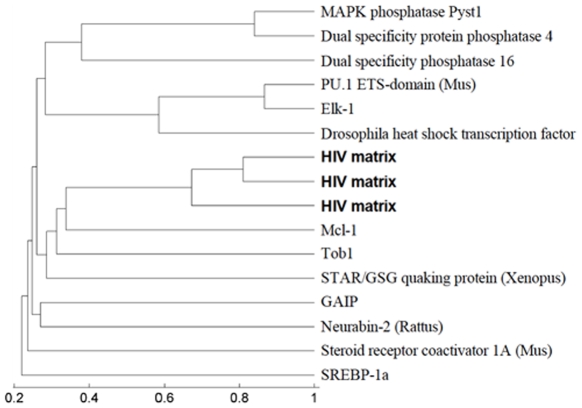
MAPK substrate hierarchy. Here we present UPGMA clustering of HIV MA proteins and ERK1/2 substrates by pairwise structural alignment TM-scores. All substrates are human proteins unless otherwise indicated. The HIV MA proteins are shown in bold.

In order to further support the feasibility of our new docking patterns as MAPK docking sites, we compared the structures of the HIV matrix protein to those of ERK1/2 substrates. We chose MA in this comparison due to the availability of multiple structures for this protein. Figure 4 shows the hierarchical clustering of the HIV MA proteins and ERK1/2 substrates (with known structures) by their pairwise structural similarity, as measured by the TM-score. As explained in the methods section, the TM-score is a normalized measure of structural similarity that ranges between 0 and 1, where a score above 0.20 is considered significant [23]. As expected by their 90% sequence similarity, the HIV MA proteins (shown in bold in Figure 4) were clustered together at a high TM-score (0.65). Surprisingly, some of the human MAPK substrates (specifically, Mcl-1, Tob1, and the Xenopus STAR/GSG quaking protein) were found to be structurally more similar to HIV MA proteins than they were to other MAPK substrates. These results were consistent with experimental data showing binding between ERK1/2 and HIV MA.

**Figure 5:**
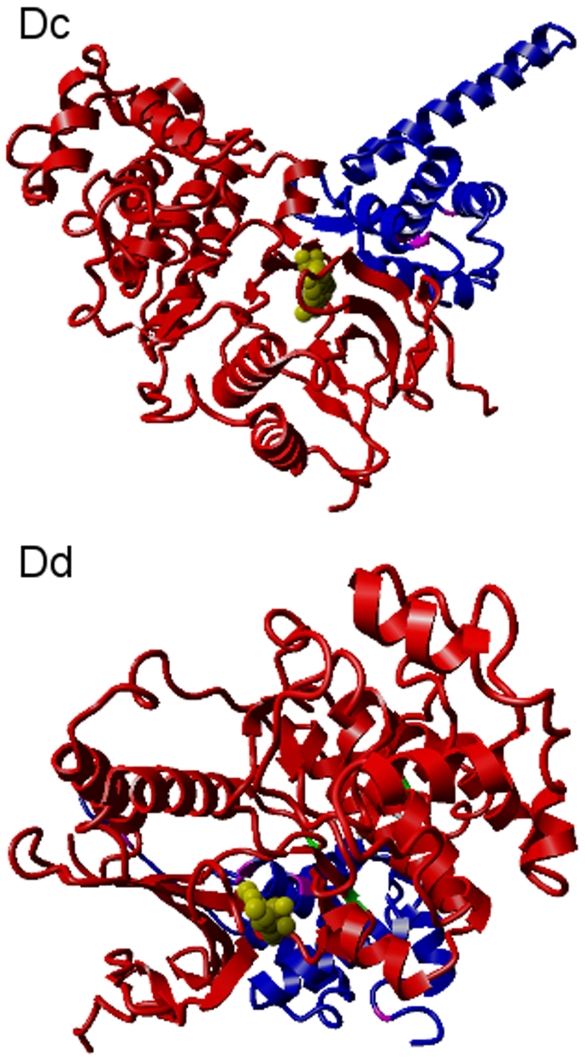
Docking between ERK1 and HIV MA. *In silico* docking was performed using ZDOCK by forcing ERK1 (orange) to dock at the *Dc* and *Dd* motifs on HIV MA (blue). In the upper panel, docking was forced to occur between the hydrophobic tail of the *Dc* motif on MA and the hydrophobic docking groove of ERK1. The lower panel shows the resulting complex when docking was forced between the basic residues of *Dd* on MA and the CD site of ERK1. The ATP binding site of ERK1 interacts with MAPK inhibitor 5-iodotubericidin (yellow). When the *Dd* docking site on MA (gray) was forced to interact with ERK1, serine phosphorylation sites (magenta) on ERK1 were positioned in close proximity to the ERK1 ATP binding site.

We next performed *in silico* docking between MA and ERK1 using the ZDOCK server [24]. ZDOCK allows users to force binding between specific residues. The top panel of Figure 5 shows ERK1 docked with MA when binding was forced between the hydrophobic portion of *Dc* and the hydrophobic groove of ERK1. The bottom panel of Figure 5 shows ERK1 docked with MA after binding was forced between the *Dd* motif on MA and the CD site on ERK1 [25]. This figure demonstrates the close proximity of the MA docking site and the ERK1 docking groove. The ATP binding site of ERK1 is bound by the MAPK inhibitor 5-iodotubericidin, colored yellow. Both docking experiments positioned possible phosphorylation sites on MA close to the ATP binding site of ERK1, adding additional evidence that the *Dc* and *Dd* patterns on MA could be functional. This is demonstrated in more detail in YASARA (http://www.yasara.org) scenes of the complexes (YASARA S1).

## Discussion

In this study we have shown that known MAPK docking motifs occur in a strain dependent manner on HIV proteins known to interact with human ERK1/2. MAPK substrate docking is known to facilitate phosphorylation. While the detailed role of MAPK phosphorylation in HIV infection has not been established in a clinical setting, ERK1/2 phosphorylation of HIV proteins has been associated with viral infectivity in a number of *in vitro* studies, highlighting the importance of MAPK docking sites in the course of HIV infection. For this reason, we hypothesized that if MAPK phosphorylation of HIV proteins was an essential feature of the progression of HIV infection, then MAPK docking sites along HIV proteins would be strain independent. This was our motivation for revising known human MAPK docking sites for the case of HIV proteins.

Our study suggested two docking motifs, *Dc*, and *Dd*, and showed that these appeared on all subtypes of HIV proteins phosphorylated by ERK1/2. These motifs shared biochemical characteristics with motifs used by human proteins that bind to MAPKs. The *Dc* motif was missing one basic residue form the standard docking motifs, while the *Dd* motif was missing the hydrophobic portion. The *Dd* motif had experimental support on human proteins, while the *Dc* motif did not. *In silico* docking experiments provided evidence supporting the hypothesis that these motifs function as MAPK docking sites along HIV proteins. One of these candidate motifs, *Dc*, was statistically enriched among the binding partners of ERK1/2. However, the lack of statistical enrichment does not exclude the possibility of the *Dd* motif being used as a docking site as well.

Current drugs target MAPK activity, but new drugs based on the HIV MAPK docking sites might work better. Existing MAPK drugs, like SB203580, SB202190, and RWJ67657 do not target ERK1/2. FR180204 does target ERK1/2 activity via the ATP binding site. New drugs targeting HIV replication by blocking ERK1/2 phosphorylation of MA and Vif could in theory be incorporated into existing therapy regimens, making it more difficult for an HIV strain to acquire the mutations for resistance to all drugs [26]. By targeting MAPK docking sites, rather than ATP binding sites, drugs can offer more specificity. There is hope that few ERK1/2 substrates will be targeted by drugs specific to HIV docking sequences. Amino acid sequences used by virus and the host in the *Dc* motif were significantly different (Figures 2 and 3) to allow for specific targeting of HIV proteins with drugs. Moreover, the poor structural alignment of HIV MA and other ERK1/2 substrates suggests that HIV specific targeting is possible.

In conclusion, the standard MAPK docking motifs from the literature could not explain ERK1/2's interactions with all subtypes of HIV proteins. The two new motifs we introduced as candidate motifs for ERK1/2 docking were present on subtypes A1, B, and C of HIV proteins known to interact with MAPK. The amino acid composition of the docking motifs on HIV proteins was different enough from the composition found on human ERK1/2 substrates to allow for HIV sequence specific drug targeting using small-molecule drugs. Further annotation of the proposed docking motifs will await experimental verification. This is partly because the sequence data used in generating regular expression patterns may contain overrepresented residues that may have no functional role in the motif, making it difficult to differentiate association from functional necessity. Present computational tools are not yet advanced enough to accurately classify functional residues in short motifs.

## Materials and Methods

### Human and HIV Sequences and Motifs

HIV sequence alignments were gathered from the LANL HIV Sequence Database, and processed according to [27], but here only subtypes A1, B, and C. We gathered 1436 proteins known to be phosphorylated from dbPTM [19], and found 132 of these were phosphorylated by ERK1/2. We scanned all sequences with the four regular expressions for MAPK docking sites. For human proteins, we attempted to rule out false positive hits in a manner similar to the ELM Resource. We removed any pattern hits that overlapped with a Pfam domain [20]. Pfam domains were found for all proteins using the default settings for the standalone Pfam scan program. Enrichment of docking site pattern hits on ERK1/2 substrates was calculated with a one-tailed Fisher's exact test, using phosphorylated dbPTM substrates as a background set.

### Structure Analysis

We performed a BLAST [28] search on the Protein Data Bank (PDB) [29] to identify known ERK1/2 substrate protein structures (E-value threshold of E-10). We collected the proteins that had less than 150 residues, to be comparable to HIV MA in size, and only kept the top hit in each BLAST result set. A pairwise structural alignment of each of three MA structures [PDB∶1hiw, PDB∶1uph, PDB∶2hmx] against each MAPK substrate structure was performed using Vorometric [30]. The alignments were filtered by a TM-score [23] of 0.25, resulting in 10 ERK1/2 substrate structures. The PDB identifiers for the 10 substrates are as follows: PDB∶1mkpA - MAPK phosphatase Pyst1, PDB∶3ezzA - Dual specificity protein phosphatase 4, PDB∶2vswA - Dual specificity phosphatase 16, PDB∶1pueE - PU.1 ETS-domain, PDB∶1duxC - Elk-1, PDB∶1hksA - Drosophila heat shock transcription factor, PDB∶2pqkA - Mcl-1, PDB∶2z15A - Tob1, PDB∶2bl5A - STAR/GSG quaking protein, PDB∶1cmzA - GAIP, PDB∶2g5mB - Neurabin-2, PDB∶1oj5A - Steroid receptor coactivator 1A, PDB∶1am9A - SREBP-1a.

### Docking

For the top panel of Figure 4, the ZDOCK server (http://zdock.bu.edu) was used to calculate the most probable complex of ERK1 [PDB∶2zoqA] and HIV MA [PDB∶1uphA] when binding was forced between the hydrophobic tail residues of the *Dc* motif on MA (Ile19 and Leu21) and the hydrophobic docking groove of ERK1 (Thr127, Leu132, Leu138, and Phe146 [25]).

For the bottom panel of Figure 4, the ZDOCK server was used to calculate the most probable complex of ERK1 [PDB∶2zoqA] and HIV MA [PDB∶1uphA] when binding was forced between the basic residues of the *Dd* motif on MA (Arg22, Lys26, and Lys27) and the CD site of ERK1 (Glu98, Asp179, Asp335, and Asp338 [25]).

## Supporting Information

YASARA S1 These are the YASARA scene files showing the ERK1/HIV MA complex determined by ZDOCK by forcing docking at the Dc or Dd motifs on MA. Molecules are colored according to Figure 5.
(0.16 MB ZIP)
